# Antiretroviral Therapy during Tuberculosis Treatment and Marked Reduction in Death Rate of HIV-Infected Patients, Thailand^1^

**DOI:** 10.3201/eid1307.061506

**Published:** 2007-07

**Authors:** Somsak Akksilp, Opart Karnkawinpong, Wanpen Wattanaamornkiat, Daranee Viriyakitja, Patama Monkongdee, Walya Sitti, Dhanida Rienthong, Taweesap Siraprapasiri, Charles D. Wells, Jordan W. Tappero, Jay K. Varma

**Affiliations:** * Ministry of Public Health, Ubon-ratchathani, Thailand; † Ministry of Public Health, Nonthaburi, Thailand; ‡ Thailand Ministry of Public Health–Centers for Disease Control and Prevention Collaboration, Nonthaburi, Thailand; § Centers for Disease Control and Prevention, Atlanta, Georgia, USA

**Keywords:** HIV, AIDS, antiretroviral therapy, tuberculosis, mortality, Thailand, research

## Abstract

Antiretroviral therapy (ART) is lifesaving in patients with advanced HIV infection, but the magnitude of benefit in HIV-infected patients receiving tuberculosis (TB) treatment remains uncertain, and population-based data from developing countries are limited. We prospectively collected data about HIV-infected TB patients from February 2003 through January 2004 in Ubon-ratchathani, Thailand. During 12 months, HIV was diagnosed in 329 (14%) of 2,342 patients registered for TB treatment. Of patients with known outcomes, death during TB treatment occurred in 5 (7%) of 71 who received ART and 94 (43%) of 219 who did not. Using multivariate analysis, we found a large reduction in the odds of death for patients receiving ART before or during TB treatment (odds ratio, 0.2; 95% confidence interval, 0.1–0.5), adjusting for CD4 count, smear status, co-trimoxazole use, and treatment facility. ART is associated with a substantial reduction in deaths during TB treatment for HIV-infected TB patients in Thailand.

## Abstract 2

Antiretroviral therapy is associated with a substantial reduction in deaths during TB treatment for HIV-infected TB patients.

Tuberculosis (TB) is one of the most common opportunistic infections and causes of death in HIV-infected persons (*1*). In developing countries, many HIV-infected persons frequently receive the diagnosis of HIV infection or AIDS after first having TB diagnosed at a health facility (*1*). The proportion of HIV-infected TB patients who die during TB treatment is high: an estimated 6%–39% die during TB treatment in sub-Saharan Africa (*2*,*3*). Deaths occurring in the first few months after TB diagnosis are more likely TB related, whereas deaths occurring later are more likely to be attributable to other HIV-related illnesses (*4*–*7*).

Thailand has experienced a severe TB/HIV syndemic, i.e., 2 diseases acting synergistically to cause excess illness and death (*8*). Almost 600,000 persons are currently HIV infected, and >90,000 TB cases are estimated to occur annually (*2*,*9*). One fourth of persons in whom AIDS was diagnosed first have TB, and an estimated 12% of TB cases in Thailand are HIV associated, although the proportion is as high as 40% in some provinces (*10*).

Antitretroviral therapy (ART), which uses highly active combinations of drugs, improves survival in HIV-infected persons (*11*,*12*). HIV-infected persons in Thailand now have widespread access to ART, but physicians often do not prescribe it to HIV-infected TB patients because of concerns about drug-drug interactions, overlapping toxicities, immune reconstitution syndrome, and pill burden. Expert groups and the World Health Organization (WHO) also recommend that public health programs make treatment of TB the first priority and ideally begin ART after TB treatment is tolerated and CD4^+^ T-lymphocyte (CD4) count is measured (*13*). Several studies have now documented that ART reduces the likelihood of death during TB treatment of HIV-infected TB patients, but these studies relied on retrospective data collection, occurred outside routine public health programs, or involved resource-rich countries without large TB or HIV epidemics (*14*–*18*). In this study, we analyzed data from a prospective, population-based surveillance system to estimate the benefit of ART on reducing mortality during TB treatment in HIV-infected TB patients living in rural Thailand.

## Methods

### Setting

Ubon-ratchathani is a large, predominantly rural province in northeastern Thailand with a population of 1.7 million persons. The rate of reported TB cases is 145/100,000 persons, and HIV prevalence in women attending public antenatal clinics was 0.6% (in 2004). Treatment of TB or HIV is offered by 25 health facilities, including 20 Ministry of Public Health (MOPH) hospitals, 3 private hospitals, 1 military hospital, and 1 MOPH outpatient TB and HIV referral clinic. Except for those who are seriously ill, TB and HIV patients are managed in outpatient specialty clinics at these facilities.

### Data Collection

In 2003, the US Centers for Disease Control and Prevention (CDC) began collaborating with the MOPH and Ubon-ratchathani Province on a special project to enhance surveillance, monitoring, evaluation, and treatment of TB, HIV-associated TB, and multidrug-resistant TB (MDR TB) in a project known as the Thailand TB Active Surveillance Network. For all patients with a diagnosis of TB in any of the 25 participating healthcare facilities, public health staff recorded standardized epidemiologic data, collected sputum specimens for laboratory testing (including staining for acid-fast bacilli [AFB], mycobacterial culture, species identification, and drug-susceptibility testing), and offered HIV counseling and testing.

Sputum specimens were collected for AFB staining and culture at the beginning of TB treatment. Specimens were cultured at an MOPH laboratory in the province on Ogawa (February–March 2003) or Lowenstein-Jensen agar (April 2003–January 2004) by using standard methods, and isolates were sent to the MOPH national reference laboratory for drug-susceptibility testing. Public health staff from the TB program collected patient data prospectively from routine medical and laboratory records, recorded data in a modified version of the standard national TB register, and entered data into an electronic database.

### Patient Population

All persons registered for TB treatment, regardless of their final diagnosis, were considered TB patients, consistent with WHO guidelines (*19*). We restricted our analysis to TB patients who had laboratory confirmation of HIV infection and who were registered for TB treatment from February 2003 through January 2004. Patient outcomes were only recorded through the end of TB treatment, which was usually 6 months after treatment initiation; no data about outcomes were recorded after the end of TB treatment.

For TB treatment, patients received standardized regimens, consistent with WHO guidelines; new (not previously treated) patients received isoniazid, rifampin, ethambutol, and pyrazinamide (*18*). HIV-infected TB patients were referred to HIV-related care and treatment, but individual physicians used their own clinical judgment about measuring CD4 count, providing opportunistic infection prophylaxis or ART, and managing other clinical conditions. When measured, CD4 counts were usually checked within the first month of TB treatment. Thai MOPH guidelines recommend that HIV-infected patients with CD4 <200 cells/mm^3^ receive co-trimoxazole and stavudine, lamivudine, and nevirapine (known as “GPO-vir”); in patients with TB, efavirenz is recommended instead of nevirapine.

### Definitions

Standard WHO definitions were used to categorize patients according to previous TB treatment history, type of TB (sputum smear–positive, pulmonary; sputum smear–negative, pulmonary; extrapulmonary), and treatment outcome. Consistent with WHO guidelines, we classified all deaths occurring during TB treatment, whether the cause was known or not, as a TB death (*18*).

We classified ART use according to whether ART was begun before TB treatment, begun during TB treatment, or not taken during TB treatment. We classified co-trimoxazole use as either taken or not taken during TB treatment. No data on interruptions of ART or co-trimoxazole treatment were collected; for the purposes of surveillance, any patient already taking or started on ART or co-trimoxazole was considered to be taking it throughout TB treatment. We stratified CD4 count (cells/mm^3^) as <50, 50–99, 100–199, and >200.

### Data Analysis

For descriptive analysis, all patients were included. For univariate and multivariate analysis of risk factors for death, we restricted our analysis to TB patients with an outcome of cured, completed, failed, or died; we excluded patients who defaulted on treatment or transferred out, because their final treatment outcome was not known. Patients with an outcome of failure were combined with those who were cured or completed treatment, since all 3 groups were known to have survived the first 6 months of TB treatment. We calculated relative risk (RR) for factors associated with death in patients with all forms of TB and in the subset of patients with pulmonary, sputum smear–positive TB.

For multivariate analysis, we calculated adjusted odds ratios (OR) for factors associated with death by using logistic regression. Variables were chosen based on >1 of the following: statistical significance (p<0.05) in univariate analysis, biologic plausibility, or previously published evidence. Because 41% of patients had data missing for CD4 count, we performed several analyses to explore the impact of missing CD4 count on our final model estimates, including the following: 1) classifying patients with unknown CD4 as a separate strata in analysis; 2) recoding patients with unknown CD4 as having CD4 <50 cells/mm^3^; 3) recoding patients with unknown CD4 as CD4 >200 cells/mm^3^; and 4) excluding all patients with unknown CD4 from the analysis. Because rates of default were high and cases of default may actually be deaths, we also recoded cases of default as death and repeated all multivariate analyses. In analyses for which there were no outcomes in some CD4 strata, we log-transformed the CD4 count and modeled it as a continuous variable (*20*). The protocol for this project was reviewed by the Thailand MOPH and CDC and the study was found to be surveillance and public health program implementation and not human subjects research requiring oversight by an institutional review board.

## Results

From February 2003 through January 2004, 2,342 patients were registered for TB treatment in Ubon-ratchathani Province. Of these, 225 (10%) were known to be HIV infected before their TB diagnosis. Of the remaining 2,117 patients, 1,626 (77%) received HIV pretest counseling, 680/1,626 (42%) agreed to HIV testing, and 104/680 (15%) were found to be HIV infected. In all, 329 (14%) of the 2,342 total TB patients were either known to be HIV infected before TB diagnosis (225; 68% of all TB/HIV patients) or were identified as HIV infected after testing through the TB program (104; 32% of all TB/HIV patients).

The median age of the 329 HIV-infected TB patients was 32 years (range 10 months–68 years), 112 (34%) were female, and 307 (93%) had new TB cases (Table 1). TB was classified as sputum smear–positive in 120 (36%), sputum smear–negative in 107 (33%), and extrapulmonary in 102 (31%). CD4 count was unavailable or not performed in 134 (41%). Of the 195 patients with CD4 results available, the median CD4 count was 53 cells/mm^3^ (range 1–873); 93% had CD4 <200 cells/mm^3^.

**Table 1:** Characteristics of HIV-infected patients with tuberculosis (TB), Ubon-ratchathani, February 2003 through January 2004

Characteristic	Patients (N = 329), no. (%)
Female	112 (34)
Median age, y (range)	32 (0.8–68)
Type, location of TB	
Sputum smear-positive, pulmonary	120 (36)
Sputum smear-negative, pulmonary	107 (33)
Extrapulmonary	102 (31)
Category of TB	
New	307 (93)
Treatment after interruption, failure, default, or relapse	11 (4)
Other	11 (3)
HIV status known before TB diagnosis	225 (68)
CD4 count (cells/mm^3^)	
<50	95 (29)
50–99	39 (12)
100–199	36 (11)
>200	25 (8)
Unknown	134 (41)
Received co-trimoxazole during TB treatment	225 (68)
Received antiretroviral therapy (ART)	
Before TB diagnosis	30 (9)
During TB treatment	45 (14)
Not prescribed ART	254 (77)
Among patients receiving ART, regimen prescribed (n = 75)	
Stavudine/Lamivudine/Nevirapine	38 (51)
Stavudine/Lamivudine/Efavirenz	35 (47)
Other	2 (2)
Treatment outcome	
Cured	61 (19)
Completed	126 (38)
Failure	4 (1)
Default	31 (9)
Transfer	4 (1)
Change of diagnosis	4 (1)
Died	99 (30)

Sputum cultures were performed in 145 (64%) of 227 patients with pulmonary TB, including 93 (78%) of 120 with sputum smear–positive TB and 52 (49%) of 107 with sputum smear–negative TB (Table 2). Of the 93 patients whose sputum smears were positive and who had a culture performed, 65 (70%) grew *Mycobacterium tuberculosis* (MTB); of these, 4 (6%) isolates were resistant to at least isoniazid and rifampin, i.e., MDR TB. Of the 52 sputum smear–negative patients with a culture performed, only 3 (6%) were culture positive, and none exhibited MDR TB.

**Table 2:** Results of culture and susceptibility testing performed on HIV-infected patients with pulmonary TB, stratified by sputum smear–positive versus –negative results, Ubon-ratchathani, February 2003 through January 2004*

Characteristic	Smear-positive (n = 120), no. (%)	Smear-negative (n = 107), no. (%)
Sputum culture performed	93/120 (78)	52/107 (49)
Culture positive for nontuberculous mycobacteria	2/93 (2)	0/52 (0)
Contaminated	5/93 (5)	6/52 (12)
Culture negative	21/93 (23)	43/52 (83)
Culture positive for *Mycobacterium tuberculosis*	65/93 (70)	3/52 (6)
Multidrug-resistant TB	4/65 (6)	0/3 (0)
Previously treated	1/4 (25)	NA

*TB, tuberculosis; NA, not available.

Before TB treatment, 30 (9%) patients were receiving ART; an additional 45 (14%) patients began ART during TB treatment; and the remaining 254 (77%) patients did not receive ART before or during TB treatment. In 40 of the 45 patients who began ART during TB treatment and in whom a date of starting ART was available, the median time between TB diagnosis and ART initiation was 93 days (range 0–170 days). Among all patients receiving ART, 38 (51%) received a combination regimen of stavudine, lamivudine, and nevirapine; 35 (47%) received efavirenz instead of nevirapine; and 2 (2%) were on other regimens. During TB treatment, 225 (68%) received co-trimoxazole.

Of all 329 patients, 187 (57%) were cured or completed TB treatment; 99 (30%) died during TB treatment. In the remaining 43 patients, treatment failed (for 4 patients) or the patient defaulted (a WHO term defined as missing at least 2 continuous months of treatment) (31 patients), transferred out (4 patients), or received a final diagnosis other than TB (4 patients). Of the 4 patients with MDR TB, 3 died and 1 was recorded as having failed treatment with final outcome not recorded.

In univariate analysis restricted to the 290 patients with an outcome of cured, completed treatment, failed treatment, or died, we analyzed several factors associated with death during TB treatment. For all TB patients, having an unknown CD4 count was associated with increased likelihood of death, and receiving co-trimoxazole or ART was associated with reduced mortality (Table 3). For ART, 5 (7%) of 71 patients who received ART died compared with 94 (43%) of 219 patients who did not receive ART (RR 0.2; 95% confidence interval [CI] 0.1–0.4; absolute risk reduction 36; number-needed-to-treat 2.8). For sputum smear–positive TB patients, results were similar; additionally, male patients were at higher risk for death than female patients (RR 2.3, 95% CI 1.1–4.7).

**Table 3:** Univariate analysis of risk factors for death among HIV-infected TB patients with outcomes of cured, completed, failed, or died, stratified by all patients versus pulmonary, smear-positive patients, Ubon-ratchathani, February 2003 through January 2004*

Characteristic	All TB patients (N = 286)		Smear-positive TB patients (n = 104)	
	Died, no./total (%)	RR (95% CI)	Died, no./total (%)	RR (95% CI)
Sex				
Male	69/193 (36)	1.2 (0.8–1.6)	33/72 (46)	2.3 (1.1–4.7)
Female	30/97 (31)	Ref	7/35 (20)	Ref
Age				
<18 y	4/19 (21)	0.6 (0.2–1.4)	0/1 (0)	0 (0–0)
18–34 y	60/168 (36)	Ref	26/60 (43)	Ref
>35 y	35/103 (34)	1.0 (0.7–1.3)	14/46 (30)	0.7 (0.4–1.2)
Type, location of TB				
Sputum smear-positive, pulmonary	40/107 (37)	Ref	NA	NA
Sputum smear-negative, pulmonary	38/96 (40)	1.1 (0.8–1.5)	NA	NA
Extrapulmonary	21/87 (24)	0.7 (0.4–1.0)	NA	NA
CD4 count (cells/mm^3^)				
>200	1/22 (5)	Ref	0/8 (0)	Ref
100–199	5/33 (15)	3.3 (0.4–26.6)	2/12 (17)	Undefined
50–99	9/35 (26)	5.7 (0.8–41.6)	1/10 (10)	Undefined
<50	22/92 (24)	5.3 (0.8–37.0)	9/34 (27)	Undefined
Unknown	62/108 (57)	12.6 (1.9–86.3)	28/43 (65)	Undefined
Co-trimoxazole during TB treatment				
Received	57/208 (27)	0.5 (0.4–0.7)	21/71 (30)	0.6 (0.4–0.9)
Did not receive	42/82 (51)	Ref	19/36 (53)	Ref
Antiretroviral therapy before or during TB treatment				
Received	5/71 (7)	0.2 (0.1–0.4)	1/23 (4)	0.1 (0.0–0.7)
Did not receive	94/219 (43)	Ref	39/84 (46)	Ref
Sputum culture†				
Culture positive	18/60 (30)	Ref	18/59 (31)	Ref
Not culture positive	81/230 (35)	1.2 (0.8–1.8)‡	22/48 (46)	1.5 (0.9–2.5)‡
Culture negative	21/60 (35)	1.2 (0.7–2.0)‡	9/20 (45)	1.5 (0.8–2.7)‡

*TB, tuberculosis; RR, relative risk; CI, confidence interval; Ref, referent; NA, not applicable. †”Culture positive” includes all patients with a sputum culture positive for *Mycobacterium tuberculosis* (MTB). “Not culture positive” includes any patients without a culture positive for MTB, regardless of whether they had a specimen sent for culture or not. “Culture negative” includes only patients with a sputum culture negative for MTB. ‡Compared with culture positive.

In multivariate analysis adjusted for CD4 count, smear status, hospital providing treatment, and co-trimoxazole use, ART remained strongly associated with reduced mortality during TB treatment (Table 4). The adjusted OR (aOR) for death in patients who received ART before or during TB treatment was 0.2 (95% CI 0.1–0.5) compared with that in patients who did not receive ART. Receiving co-trimoxazole was no longer significantly associated with reduced mortality. We found virtually identical results when we did the following: 1) restricted our analysis to only those patients who received ART during TB treatment compared with patients who did not receive ART during TB treatment; 2) restricted our analysis to previously untreated, non-MDR patients without nontuberculous mycobacteria; 3) coded patients with unknown CD4 as having CD4 >200 cells/mm^3^, as having CD4 <50 cells/mm^3^, or as missing (i.e., removed from the analysis). All analyses also produced essentially identical results when we reclassified cases of default as death.

**Table 4:** Multivariate analysis of risk factors for death among HIV-infected TB patients with outcomes of cured, completed, failed, or died and adjusted for site of treating facility,* Ubon-ratchathani, February 2003 through January 2004†

Characteristic	Adjusted OR (95% CI)
Antiretroviral therapy before or during TB treatment	0.2 (0.1–0.5)
Co-trimoxazole during TB treatment	1.1 (0.6–2.3)
CD4 count (cells/mm^3^)	
>200	Referent
100–199	5.5 (0.6–52.6)
50–99	9.3 (1.0–82.9)
<50	9.7 (1.2–78.4)
Unknown	29.9 (3.8–238.0)
Type, location of TB	
Sputum smear–positive, pulmonary	Referent
Sputum smear–negative, pulmonary	1.3 (0.7–2.6)
Extrapulmonary	0.5 (0.2–1.0)

*Data not shown. †TB, tuberculosis; OR, odds ratio; CI, confidence interval.

When we restricted our analysis to sputum smear–positive patients, we found a similarly strong beneficial effect for ART. Because no deaths occurred in the group of smear-positive patients with CD4 >200 cells/mm^3^, we modeled CD4 as a continuous variable. The aOR was 0.1 (95% CI 0.0–0.9). Results were similar when we recoded patients with unknown CD4 count as having CD4 equal to 50 cells/mm^3^ (indicative of profound immunosuppression and imminent risk of death) or 250 cells/mm^3^ (not eligible for antiretroviral treatment in many country guidelines because they are relatively immune competent). Because of small sample size, we were only able to perform univariate, not multivariate, analysis for the 57 culture-positive patients who had an outcome of cured, completed, failed, or died. One (8%) of 13 culture-positive patients receiving ART died compared with 17 (36%) of 47 culture-positive patients not receiving ART (RR 0.2, 95% CI 0.0–1.5; absolute risk reduction 28; number-needed-to-treat 3.6).

Because patients who died soon after TB diagnosis were also unlikely to have begun ART, we modeled the effect of ART after excluding 32 patients who died in the first month of beginning TB treatment (aOR 0.1, 95% CI 0.0–0.8) and the additional 16 patients who died in the second month (aOR 0.1, 95% CI 0.0–1.2). Results were similar when we recoded patients with unknown CD4 count as having CD4 equal to 50 cells/mm^3^ or 250 cells/mm^3^, except that the effect of ART was now statistically significant for the analysis excluding deaths within the first and second months (aOR 0.1, 95% CI 0.0–0.7 for unknown CD4 re-coded as 50 cells/mm^3^; aOR 0.1; 95% CI 0.0–0.9 for unknown CD4 recoded as 250 cells/mm^3^).

We explored the effect of co-trimoxazole on mortality for the 218 patients who did not receive ART: 52 (38%) of 137 patients receiving co-trimoxazole died compared with 42 (51%) of 82 patients not receiving co-trimoxazole (RR 0.7, CI 0.6–1.0; absolute risk reduction 13; number-needed-to-treat 7.7). The association between co-trimoxazole and survival was not statistically significant when we excluded patients who died in the first month (RR 0.9, CI 0.5–1.5) or in the first 2 months (RR 1.2, CI 0.5–3.0) and when we limited the analysis to smear-positive patients (RR 0.8, CI 0.5–1.3). In multivariate analysis of patients who did not receive ART and adjusting for CD4 count, smear status, and hospital providing treatment, co-trimoxazole was not associated with survival (aOR 0.9, CI 0.5–1.9).

## Discussion

In this prospective, population-based study from a rural province in northeastern Thailand, we documented a high rate of death in HIV-infected TB patients and a substantial reduction in the risk for death during TB treatment for patients receiving ART. In this population, TB occurred predominantly in persons with preexisting HIV diagnoses and low CD4 counts, and, as expected, the CD4 count was inversely related to death. These findings are more consistent with the epidemiology of TB in high-income countries, such as the United States, than that in sub-Saharan Africa, where studies have found that most TB and HIV have not previously been diagnosed in patients with HIV infection and that TB occurs across a broad spectrum of immune suppression (*21*–*26*). Although CD4 counts were not recorded for many patients in this study, we can infer from the high mortality in these patients that their CD4 counts were probably low. Research would be needed to document whether data from this evaluation are representative of other settings in Thailand or Southeast Asia. That TB occurred predominantly in persons with advanced, diagnosed HIV infection suggests that interventions targeted specifically at HIV-infected patients—such as early ART, treatment of latent TB infection, and earlier screening for and treatment of TB disease in household contacts of TB patients and during routine HIV care—are also needed to reduce incidence and mortality of HIV-associated TB.

Given the advanced immune suppression in this population, that ART improved survival during TB treatment is not surprising. The magnitude of benefit, however, was substantial. Treating 3 HIV-infected TB patients with ART in this population would translate into 1 life saved during TB treatment. In fact, co-trimoxazole, which is known to save lives during TB treatment in Africa (*27*), was not significantly associated with survival after adjusting for ART use. Co-trimoxazole protects AIDS patients against a wide range of infections that commonly occur in Thailand, including *Pneumocystis jirovecii* and *Toxoplasma* sp. (*28*). We were not able to demonstrate a survival benefit of co-trimoxazole in patients receiving ART or those not receiving ART. We do not know whether this is a true phenomenon or attributable to bias, misclassification, or small sample size. Further studies are needed to evaluate the survival benefit of co-trimoxazole in HIV-infected TB patients in Thailand. Our sample size was too small to compare outcomes between patients receiving regimens with efavirenz versus nevirapine or to compare outcomes between patients who received ART during the first 2 months of TB treatment compared with those who received ART later.

Our evaluation reinforces the importance of providing TB patients with early access to HIV diagnosis and treatment, as recommended in WHO’s Interim Policy on TB/HIV Collaborative Activities (*1*). In this project, we found several missed opportunities, including HIV testing of TB patients, measurement of CD4 count, and initiation of co-trimoxazole and ART. Educating providers about the life-saving benefits of ART in HIV-infected TB patients is a major priority, but more data from observational studies and clinical trials are needed to provide evidence-based guidance about the optimum timing of ART and the incidence and management of overlapping toxicities and immune-reconstitution syndrome.

This study has several major limitations. First, we do not know the reasons why patients did not receive ART. Many patients who did not receive ART were likely deemed too ill and, therefore, unlikely to benefit from ART. This bias would exaggerate the benefit of ART; survival would determine ART use, not the reverse. To address this issue, we controlled for 2 factors likely to predict when a patient would receive ART, such as CD4 count and hospital of care (a surrogate for physician preference or resources). Moreover, after excluding persons who died within the first 2 months of TB treatment, i.e., persons likely to receive minimal benefit from ART and likely to have been deemed too ill to receive ART, we still identified a substantial benefit for ART.

Another limitation is that not all TB patients underwent HIV testing, which could skew our population toward those patients most likely to have advanced immune suppression and, therefore, most likely to benefit from ART. Since these data were collected, rates of HIV testing of TB patients have increased substantially in Ubon-ratchathani, but the proportion identified as HIV infected remains similar to what it was in this study, suggesting that the number of HIV infections missed in this study population was small. This study was also based on surveillance and monitoring data from a public health program, which, though prospectively collected, necessarily relied on incomplete data, such as from routine medical records. Core data elements, such as CD4 count, were missing for many patients, and data about adverse events and causes of death, which are critical to assessing the risks of combined ART and TB treatment, were not collected. Low rates of culture positivity, particularly in smear-negative patients, leave open the possibility that some patients did not, in fact, have TB. Since this study, we have identified several reasons for the low yield of culture, including delayed transport times for specimens and inadequate specimen collection; efforts to improve these procedures have since been implemented. Even though many patients were not culture confirmed in this study, sputum smear–positive patients benefited strongly from ART, which suggests that misdiagnosis of TB is an unlikely explanation for our findings.

Major strides have been made in enhancing access to HIV treatment in the developing world. Nevertheless, as this study shows, deaths of patients with both TB and HIV remain high, and, even in a country such as Thailand with high rates of access to ART, few HIV-infected TB patients receive ART. Globally, measures to save lives of patients with both diseases have focused on making TB and HIV programs collaborate more closely. To that end, more data from these settings are urgently needed to convince policymakers in countries affected by this syndemic about the critical importance of rapidly expanding access to ART, particularly for HIV-infected TB patients.
